# Non-Linear Neuronal Responses as an Emergent Property of Afferent Networks: A Case Study of the Locust Lobula Giant Movement Detector

**DOI:** 10.1371/journal.pcbi.1000701

**Published:** 2010-03-12

**Authors:** Sergi Bermúdez i Badia, Ulysses Bernardet, Paul F. M. J. Verschure

**Affiliations:** 1 SPECS - Laboratory for Synthetic Perceptive, Emotive and Cognitive Systems, Universitat Pompeu Fabra, Barcelona, Spain; 2 ICREA - Institució Catalana de Recerca i Estudis Avançats, Barcelona, Spain; Université Paris Descartes, Centre National de la Recherche Scientifique, France

## Abstract

In principle it appears advantageous for single neurons to perform non-linear operations. Indeed it has been reported that some neurons show signatures of such operations in their electrophysiological response. A particular case in point is the Lobula Giant Movement Detector (LGMD) neuron of the locust, which is reported to locally perform a functional multiplication. Given the wide ramifications of this suggestion with respect to our understanding of neuronal computations, it is essential that this interpretation of the LGMD as a local multiplication unit is thoroughly tested. Here we evaluate an alternative model that tests the hypothesis that the non-linear responses of the LGMD neuron emerge from the interactions of many neurons in the opto-motor processing structure of the locust. We show, by exposing our model to standard LGMD stimulation protocols, that the properties of the LGMD that were seen as a hallmark of local non-linear operations can be explained as emerging from the dynamics of the pre-synaptic network. Moreover, we demonstrate that these properties strongly depend on the details of the synaptic projections from the medulla to the LGMD. From these observations we deduce a number of testable predictions. To assess the real-time properties of our model we applied it to a high-speed robot. These robot results show that our model of the locust opto-motor system is able to reliably stabilize the movement trajectory of the robot and can robustly support collision avoidance. In addition, these behavioural experiments suggest that the emergent non-linear responses of the LGMD neuron enhance the system's collision detection acuity. We show how all reported properties of this neuron are consistently reproduced by this alternative model, and how they emerge from the overall opto-motor processing structure of the locust. Hence, our results propose an alternative view on neuronal computation that emphasizes the network properties as opposed to the local transformations that can be performed by single neurons.

## Author Summary

The tiny brains of insects of about 1mm^3^ smoothly control a flying platform while avoiding obstacles, regulating its distance to objects and search for objects of interest. This is largely achieved through a complex hierarchical processing of signals from the multitude of ommatidia in their eye to a set of highly specialized neurons that are optimized to respond to specific properties of the visual world. One of these neurons, the Lobula Giant Movement Detector (LGMD) of the locust, has been recently shown to perform a functional multiplication of its synaptic inputs. If true, that would make the LGMD neuron a unique and highly sophisticated neuron that raises questions about the non-linear operations other neurons in other neuronal systems would be able to perform. Hence it is crucial to understand its properties, its role in behaviour and to evaluate whether its responses can be explained in simpler terms. Our results emphasize the role of network architecture and distributed computation as opposed to local complex non-linear computation. We show that our model reliably reproduces the known properties of the LGMD and can be used to control a high-speed robot.

## Introduction

Since the introduction of the neuron doctrine about 100 years ago, a central question has become what local operations the primitive elements of nervous systems can perform. So far, the only operation that has clear experimental support is the threshold operation that converts the depolarization of the membrane into action potentials. However, also other local non-linear operations such as multiplications and divisions have been proposed. For instance, the Elementary Motion Detector (EMD), a well-established model of motion detection in the fly visual system that relies on multiplication in order to explain the neural responses of the Horizontal and Vertical System (HS, VS) visual interneurons [1]. In addition, it has been proposed that attentional modulation can result in a multiplicative gain of neuronal response to sensory stimuli [2]. Another example is the divisive inhibition that is assumed to underlie some of the non-linear adaptation properties of cortical neurons [3],[4], while several other studies have investigated how neuronal noise or dendritic saturation could contribute to divisive gain control [5],[6]. Moreover, theoretical studies on neocortical pyramidal cells have suggested that multiplicative dendritic integration could account for non-linear sensory processing enhancing stimulus classification [7],[8]. Despite the above examples, its computational attractiveness and the fact that some data can be satisfactorily described in non-linear terms, it remains unclear how the biophysics of single neurons could implement these operations.

One particular case in point is the Lobula Giant Movement Detector (LGMD) visual interneuron of the locust. Recently it has been shown that the responses of this visual interneuron can be explained in terms of a local product of two high-level features of visual stimuli, their angular size and angular speed by means of a non-linear transfer function of the neuron [9],[10]. If correct, this is the most explicit case reported in the experimental literature that supports the notion of local non-linear neuronal operations and it will have important consequences for our understanding of the computations that the nervous system can perform, as it significantly increases the computational power we can ascribe to single neurons. Hence, given the implications of this finding, it is important to investigate whether the non-linear relationship between the responses of the LGMD neuron and the visual stimuli it is exposed to can be understood in alternative terms, yet consistent with our current knowledge of the system. Here we investigated the alternative hypothesis that the non-linear responses of the LGMD can be explained in terms of an *emergent non-linear operation* that results from the integration of distributed computations performed by the neurons of the processing architecture as a whole as opposed to being a multiplication operation that is local to a single unit, i.e. the LGMD.

**Figure 1:**
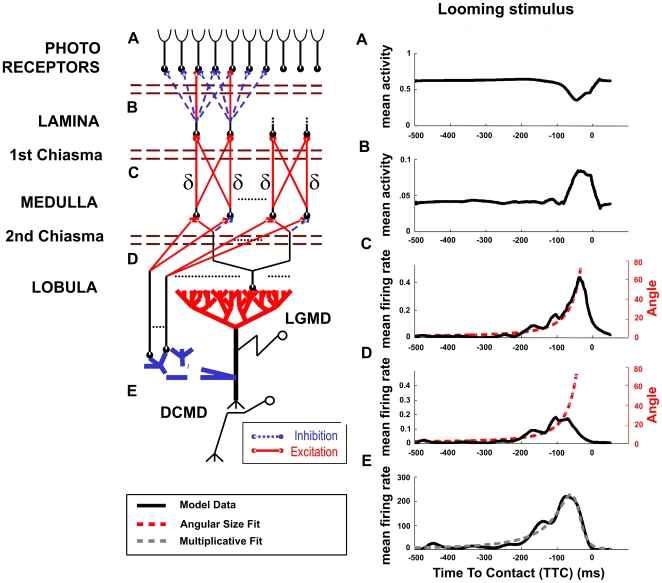
Model of the locust LGMD system. Left column depicts the modelled anatomical organization of the pathway to the LGMD (A–E) while the right column indicates the physiological responses to a looming stimulus in terms of the mean population activity averaged over 25 repetitions for each layer of the model. (A) is the photoreceptor layer, (B) a centre/surround architecture in the lamina, (C) the on-off neurons of the medulla, (D) the neurons connecting to the excitatory pathway of the LGMD, and (E) the LGMD/DCMD output. The data was fitted (dashed line) with the instantaneous angular size of the object in (C) and (D), and with the multiplicative model of the LGMD proposed by Gabbiani et al. (1999) in (E).

The LGMD neuron is a wide-field neuron that is known to respond preferentially to looming stimuli [11],[12]. Initially, it was first thought to be an on-off neuron due to its integration of neuronal responses generated in the afferent medulla layer that correlate with the onset and offset of local visual features [13]–[15]. More recently the relationship between properties of looming stimuli and the firing rate of the LGMD have been extensively documented, including the non-linear relationship between firing rate and time to collision (TTC), the constant relation between peak firing rate and angular size, the independence of the peak firing rate of the stimulus speed, shape and texture, and the linear relationship between the TTC of the LGMD peak firing rate and the apparent looming stimulus' speed [9], [16]–[18]. The LGMD has been the target of a number of theoretical studies that either investigated its collision detection capabilities [19]–[22], or its putative non-linear integration properties [9], [16]–[18]. The first model was published in the late 90's [22]. Rind et al. have shown that the integration of on- and off-channels by a LGMD model can account for aspects of its looming sensitivity and subsequently this model has been applied to collision avoidance by roving robots [22]–[26]. Recently, it has been shown that all of the known response properties of the LGMD can be accounted for in terms of the multiplication of the angular velocity (θ′) with the angular size (θ) of a looming stimulus [9], where θ and log (θ′) are directly conveyed to the LGMD via separate inhibitory and excitatory pathways (Figure 1). The membrane potential (Vm) deflection is subsequently assumed to be proportional to this multiplication that is subsequently expressed in a firing rate, *f^−l^*, via an exponential mapping:
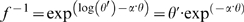
(1)where,
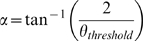
(2)and *θ_threshold_* is an animal and species dependent parameter that specifies the approaching object's angular size at which the LGMD firing rate is maximal [27]. Hence, by performing an exponential on the summed inputs an effective multiplication occurs. This model indeed provides for an excellent fit of the LGMD responses to looming stimuli, and as such constitutes a useful benchmark for any model of the LGMD [10]. Nevertheless, this local multiplicative model makes a number of strong assumptions and overlooks the role of the neurons pre-synaptic to the LGMD. More concretely: how does the fan-in to the LGMD delineate an “object” of which θ′ and θ can be assessed, given that an “object” has been defined, how are log (θ′) and θ computed, how is this high-level information represented by the massive fan-in to the LGMD, and how are the parameters related to the approaching stimulus (θ′ and θ) extracted and conveyed to the LGMD in the early visual system of the locust? Moreover, this proposal assumes that the excitatory and inhibitory inputs to the LGMD respond to high level information about the visual stimulus (θ′ and θ) and that the role of the LGMD is to compute a functional multiplication on those. By definition, the functional multiplication attributed to the LGMD heavily depends on having the two above mentioned features reliably computed and delivered to distinct pathways. However, in mathematical terms, there is not a unique combination of input signals to the LGMD that could give rise to the above described firing rate pattern (eq. 1), and thus, no reason to exclude this possibility. Indeed, our model suggests that this is the case (Figure 1, layers C–D right panel). Would the LGMD in that case still perform a functional multiplication or just a non-linear mapping of its inputs? In fact, the putative multiplicative properties of the LGMD have already been a matter of debate [28]–[30]. In this study we approach the above mentioned points from a system and architectural point of view. We evaluate the alternative hypothesis that the non-linear relationship between the responses of the LGMD neuron and the stimuli the organism is exposed to result from the interaction of many neurons in the sensory processing architecture, i.e. it is an emergent non-linearity that is read-out by the LGMD. In particular, we will assess the contribution of each processing layer in the visual processing hierarchy of the locust, how and what information is conveyed to the LGMD, and the resulting integration at the LGMD level. The empirical validation of this alternative hypothesis, however, is currently unpractical since it requires simultaneous in-vivo measurements from large numbers of neurons under well-controlled behavioural conditions. Hence, to assess the validity of our alternative “emergent non-linearity” hypothesis we resort to a computational approach and use a computational model that is consistent with the anatomy and physiology of the locust visual processing hierarchy, including the ommatidia, medulla, lobula, LGMD and the Descending Contra-lateral Movement Detector (DCMD). Using this model we show that all properties of the LGMD neuron that can be described in terms of a local non-linear operation can be explained as emerging from the structure of the network as a whole. Above all, we show that the inputs to the LGMD are directly driven by the stimulus dynamics rather than resulting from a process of segmentation or computation of the speed of the approaching objects. Despite the differences with Gabbiani's et al. model, the model proposed here displays identical responses to its biological counterpart on all standard stimulation protocols reported in the literature. We demonstrate that the emergent non-linear operations are strongly dependent on the details of the synaptic organization of the locust's visual system. In addition, we apply our model to a high-speed mobile vehicle and show that it reliably stabilizes the movement trajectory and robustly avoids collisions. Hence, our model not only suggests that the functional non-linear response properties of the LGMD emerge out of the network as a whole but also shows robust and realistic real-world properties.

## Results

### Model

The structure of our model consists of four layers that capture the most relevant processes involved in the pathway to the LGMD, and both the output of the LGMD and the population responses for each layer are considered (Figure 1). We model the photoreceptor layer with Linear Threshold (LT) units that are driven by a CCD camera with automatic gain control (see Experimental Procedures) (Figure 1A). The lamina is modelled with a centre/surround connectivity that produces an edge enhancement [31] (Figure 1B). Subsequently, neurons in the medulla layer produce onset and offset sensitive responses [13]–[15] (Figure 1C). The connectivity between the medulla and the lobula layer transduces the excitatory input to the LGMD (Figure 1D). Post-synaptic inhibition onto the LGMD is modelled through the integration of the activity of the onset and offset sensitive neurons in the medulla where the summed activity inhibits the excitatory projections onto the LGMD from the second chiasma. The LGMD is modelled as an Integrate and Fire (I&F) neuron that integrates the above mentioned excitatory and inhibitory inputs from the medulla and produces spikes (Figure 1E). All neurons in our model are standard leaky I&F or leaky LT neurons [32],[33] (see Experimental Procedures for model details and dynamic equations).

In the context of this study, we present an exhaustive analysis of the responses of our model to a set of standard LGMD stimulation protocols that allow us to validate our model with respect to the biological system. Additionally, the contribution of each neural layer of the model to the LGMD responses' properties is assessed experimentally (Figure 1A–E) as well as analytically, and its real-world properties are evaluated using a fast moving robot.

In our first experiment we evaluate the model by using a looming stimulus consisting of a solid square with 10 to 21 repetitions performed per each l/|v| pair (where l stands for the half length of the object and v for its linear velocity) (see the Experimental Procedures for further details). This ratio determines the time course of the angular size (θ) of the looming stimulus in an independent fashion from the actual stimulus properties (eq. 3). This experiment replicates the protocol used in [17].

**Figure 2:**
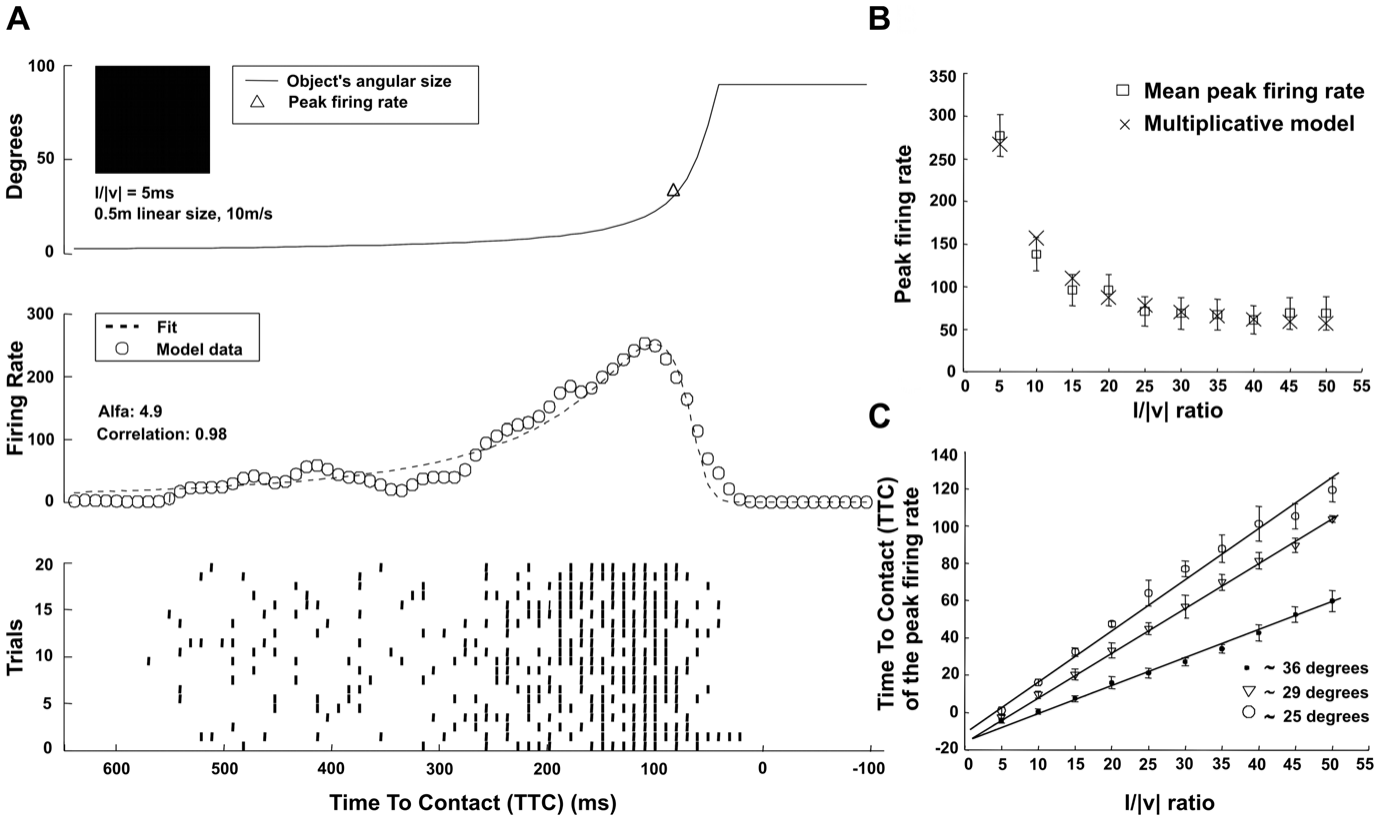
Responses of the LGMD model to a looming stimulus (solid square). (A) Upper panel: Stimulus' angular size versus time. The looming stimulus shape and parameters are indicated on the top-left corner of the graph. Middle panel: firing rate of our model over time (circles) and the corresponding fit by the multiplicative model derived from LGMD recordings (dashed line). The fit parameters and the obtained correlation values are indicated. Lower panel: Raster plot of the responses of our model over 20 trials. (B) Fit of the Peak Firing Rate of the LGMD model with eq. 1 versus the l/|v| ratio of the stimulus. (C) Time To Collision (TTC) versus the l/|v| ratio and its linear regression. The experiments were done for three different model parameters, i.e. angular threshold values, to show how this parameter affects the peak firing rate. A total of 10 experiments per l/|v| ratio and condition were performed (N = 300). Error bars indicate data variance.

Our model of the LGMD displays the typical response of this neuron to an approaching stimulus (Figure 2A); as the angular size of the retinal projection of the stimulus increases, the firing rate increases, peaks and decays before the collision occurs. This response closely resembles that of the biological data with the multiplicative model (r = 0.98) (Figure 2A, middle panel). We observe that the fit of the peak firing rate and the TTC versus the l/|v| ratio is consistent with that observed in the biological system, and is well captured by the multiplicative model that was derived from LGMD recordings (eq. 1) (Figure 2B).

The response of the LGMD neuron has been shown to peak when the angular size of the projection of the looming stimuli onto the retina of the insect reaches a specific size, known as the angular threshold [9],[10],[16],[17],[34],[35]. Moreover, the time at which the response of the LGMD peaks, that is, when the stimulus reaches the angular threshold, depends linearly on the l/|v| ratio. This reflects a robust detection of the angular threshold over a wide range of l/|v| ratios since the time at which the response of the LGMD peaks is proportional to l/|v|. The linear relationship between TTC of the peak firing rate and the l/|v| ratio is a known property of the LGMD [17],[18] (eq. 2), that is reliably replicated by our model (r>0.99) (Figure 2C).

We propose a specific connectivity for the LGMD pre-synaptic fan-in such that the projections from the medulla to the lobula integrate oriented contrast boundaries (see Experimental Procedures). These projections are retinotopic and integrate the activity of a set of on-off neurons of the medulla that surround its location at distances δx and δy (surround excitation). Consequently, δx and δy define the width and height of the region within which the boundaries of a looming stimulus have to fall in order to achieve maximal excitation, what defines the angular threshold. To further test this aspect of the model, we performed a control experiment in which we varied δx and δy to define a surround receptive field of 25, 29 and 36 degrees of the camera's field of view. The predicted behaviour of our model is that the changes in δx and δy would affect the angle of the peak firing rate, and therefore the TTC. Indeed, we obtained a change of the slope of the linear regression between the frequency peak and the l/|v| ratio which correlates with the changes in δx and δy; the bigger δx and δy, and hence the angular threshold. The later the LGMD response firing rate reaches its maximum and the flatter the slope is (Figure 2C). In conclusion, our model is consistent with the known properties of the LGMD [9],[16],[17] and shows that the response peak is defined by the topology of the projections from the medulla to the LGMD.

**Figure 3:**
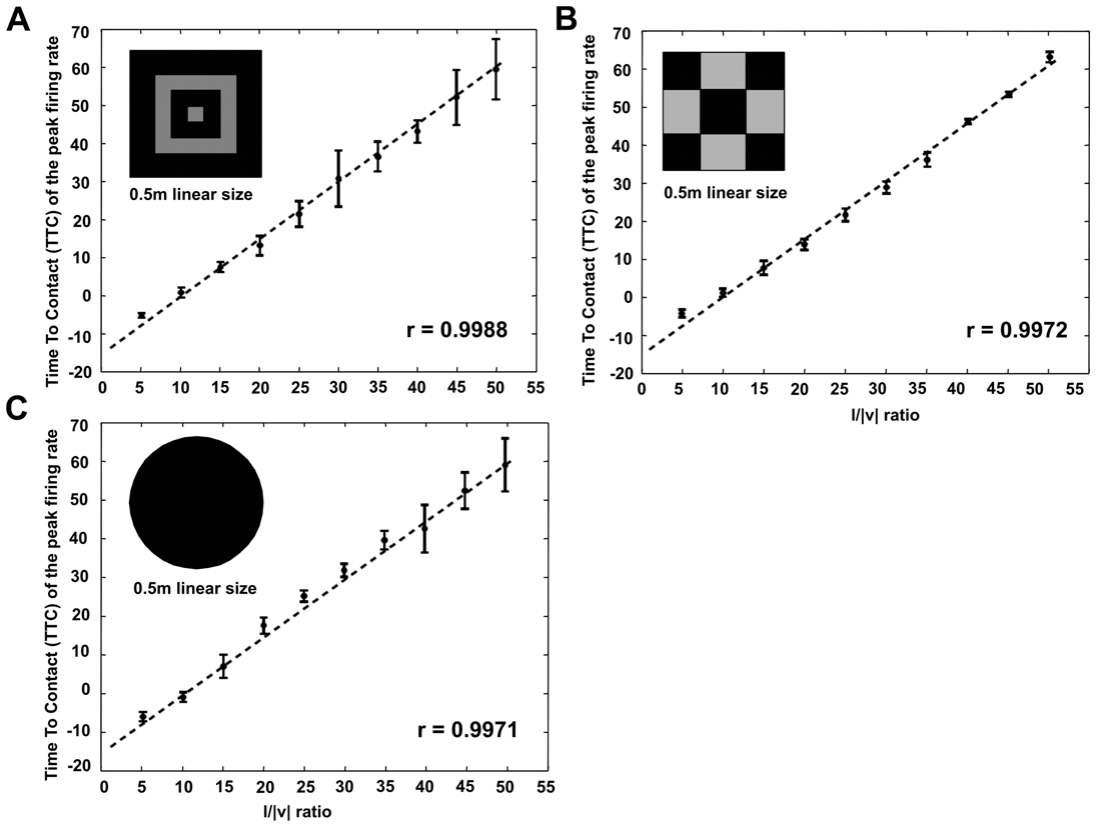
Analysis of shape and texture effect on the peak firing rate relative to the Time To Contact (TTC). (A) to (C): TTC versus the l/|v| ratio is invariant to stimulus shape and texture for the entire set of l/|v| ratios; (A) concentric squares, (B) checkerboard texture, (C) solid circular stimulus. The error bars indicate data variance; r is the correlation coefficient of the data with its linear regression.

It was shown that the responses of the LGMD are largely independent of the shape of the stimulus and its texture [17]. In a series of experiments, we assessed whether our model shows similar invariant properties (Figure 3). To do so, consistent with previous experiments [17], we used four different shapes. For all stimuli tested, and over the whole range of l/|v| ratios (from 5ms to 50ms), the model's responses show the same linear relationship with the TTC as reported for the biological system, with a correlation coefficient between the model's responses and the regression lines of r>0.99 (Figure 3C).

**Figure 4:**
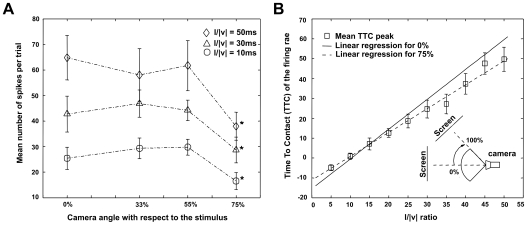
Responses of the model for different approach angles of the looming stimulus. (A) The mean number of spikes per trial is calculated for four different approach angles. At least 20 repetitions have been performed per angle/speed pair. The l/|v| ratio was varied from 10ms (circles), to 30ms (triangles) and 50ms (diamonds). (B) The difference of the TTC of peak firing rate with respect to the l/|v| ratio between a frontal approaching trajectory and one at an angle representing 75% of the visual field. Error bars indicate data variance.

The response invariance to the approach angle of looming stimuli is biologically highly relevant in a system that can serve to detect potential predators, as is the LGMD. This reported property of the LGMD was investigated in the last set of experiments. The invariance was assessed by using the same experimental protocol as previously employed, but now aligning the camera at different angular orientations with respect to the projection screen as was reported in [17]. In the following we refer to 0% of the visual field when there is a complete alignment of the camera orientation and screen, and to 100% when the centre of the screen is at the edge of the camera's visual field (Figure 4B, insertion).

We found that our LGMD model shows a robust response invariant to the approach angle up to an angle that represents approximately 75% of the visual field (Figure 4). A one-way ANOVA analysis of the distribution of the model responses revealed that a significant difference in the mean number of spikes only occurs at an angle exceeding 75% of the total visual field of the camera (approximately 30°) (p<0.05), i.e. when the stimulus was partially lying outside of the visual field of the camera. Although the fields of view of the locust eye and our camera are not equivalent, yet we have designed it to have a similar angular resolution of 2.33° per pixel [36]. Additionally, the fraction of field of view where the response is invariant is comparable to the one of the biological system [16] (Figure 4A).

Subsequently, we investigated the linear relationship of the TTC of our LGMD model over a wide range of l/|v| ratios and approach angles. Our results show that the invariance of the response properties can be seen as well in the TTC domain (Figure 4B). Here, the correlation coefficients of the data and its linear regression are above 0.9 for both a perfect alignment between the camera and the screen and in case of a misalignment of 75% of the visual field. Thus, even though the activity of the neural model is significantly reduced due to the loss of stimulation by the looming stimulus at a very shallow approach angle (Figure 4A), the intrinsic linear dependence of the TTC with respect to the l/|v| of the LGMD is preserved (Figure 4B).

### Predictions

**Figure 5:**
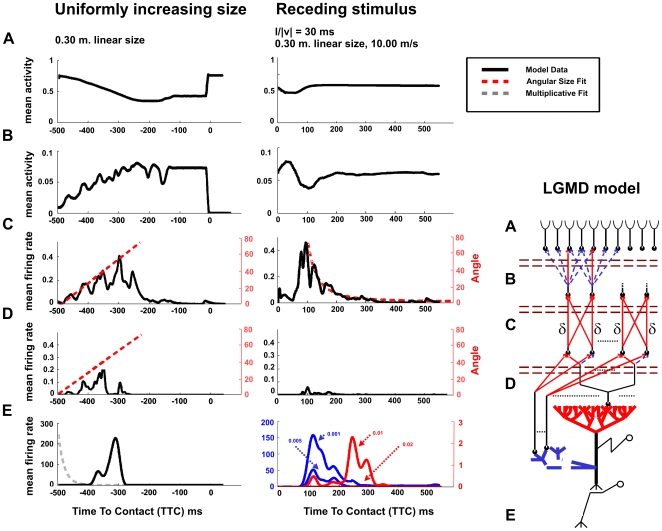
Mean population responses of the different layers of our model to a uniformly expanding or receding stimulus. The right most panel depicts the modelled anatomical organization of the pathway to the LGMD (A–E) where (A) is the photoreceptor layer, (B) the lamina (the centre/surround architecture), (C) the medulla (containing the on-off neurons), (D) the neurons connecting to the excitatory pathway of the LGMD, and (E) the LGMD/DCMD output. Left panel: Average population response for each of the layers of our model (depicted in the right most diagram) to an object that is uniformly increasing in size (10 repetitions). The curves in (E) show the different responses predicted by our model (solid line) and generated by the model proposed by Gabbiani et al. (1999) (dashed grey line) to the same stimulus. Right panel: Population responses for each of the layers of our model to receding stimuli (32 repetitions). The gain of the excitatory input to the LGMD was fixed to 0.2 while 4 different gains of the inhibition were tested (0.02, 0.01, 0.005, and 0.001). (E) shows the 4 predictions for the responses of the LGMD depending on the inhibitory weight to a receding stimulus. The input data to the LGMD was fitted with the instantaneous angular size of the object in (C) and (D) (red dashed line). The half-length of the objects for both linearly increasing and receding stimulation experiments was fixed to 30cm. In the case of the receding experiment, the simulated object was moving away from the camera at 10m/s.

In order to understand better and to be more specific about the nature of the inputs to the LGMD, we propose the use of additional stimulation protocols that can be applied to the locust using currently available experimental technologies. For instance, in the multiplicative model, the firing rate of the LGMD is defined by the product of the angular speed (θ′) and a value related to the object's angular size (θ) (eq. 1). If those two variables were indeed the input to the LGMD, it would imply that for an object that is approaching at a constant angular speed the LGMD should display a completely different time response. In fact, since the angular approaching speed of the stimulus (θ′) would be constant, the predicted output by the multiplicative model would be an exponentially decreasing firing rate. Hence, we explicitly evaluate the different responses between our model for each neural layer and the multiplicative one by using objects that show a uniform increase in size (Figure 5, layers A–E left panel). We observe that, whereas the multiplicative model displays the expected exponential decreasing response, our emergent non-linearity model still displays a peak at the preferred angular size. This stimulation protocol was previously used by Hatsopoulos et al. showing a response profile consisting of a fast increase of the firing rate, a peak and subsequently followed by a slower decrease of the activity [18]. Although some of the data could eventually be approximated by an exponential function, a more quantitative analysis of the LGMD responses is required in order to find the relationship between stimuli and rising, peak and decay properties of the responses of the LGMD under this protocol. Additionally, we see that the predicted excitatory input to the LGMD with our model differs from the constant factor predicted by the multiplicative fit (Figure 5, layer D left panel). Thus, a further examination of the LGMD responses under this protocol is essential to unveil what the real input to the LGMD is, and therefore to understand whether it computes a product of the object's angular size (θ) and angular speed (θ′) or responds to a different processing as suggested by our results.

Next, we analyze the activity of each layer of our model to identify the relationship between receding stimuli and the intensity of the LGMD response (Figure 5, layers A–E right panel). The responses are consistent with a number of experiments of stimulus selectivity of the LGMD that showed a preference for looming stimuli and its diminished response to receding ones [11],[12],[35]. Two hypothetical peaks in the TTC curve to receding stimulus are predicted depending on the weighting of the post-synaptic inhibition (Figure 5, right panel). We show that the specific amplitude-time course of this response depends on the gain of the inhibitory projections onto the LGMD.

### Robot experiments

So far we have shown that we can account for all known aspects of the responses of the LGMD neuron to looming stimuli with a model that relies on the transformations performed in the complete pathway from the photoreceptors to the LGMD as opposed to a local multiplication. We now want to assess the behavioural validity of our model by applying it to a high-speed impeller driven based robot called “Strider”. Given its structure, the Strider is highly sensitive to inertia and friction forces, yet it delivers high-speeds. For the robot to be sensitive to shallow approach angles, its camera was equipped with a wide angle lens (190 deg. field of view). Although the aim of the robot is to have dynamics comparable to that of a flying insect, our robot has longer reaction times due to its increased mass, i.e. it operates at a higher Reynolds number than a flying insect. We therefore use a course stabilization system to guarantee that the robot is able to follow straight trajectories. This course stabilization system is based on the fly's Elementary Motion Detectors (EMD) and uses directional motion information from the visual input to correct for drifts, and has been previously deployed on flying vehicles [37].

The real-world behavioural task of the robot is to drive forward on a straight course until an imminent collision is detected. The modelled LGMD neuron will detect this upcoming collision and induce a collision avoidance reaction that consists of two phases: first deceleration of the robot, and then change of heading direction. To deal with the inertia of the robot, the braking manoeuvre is realized by driving the impellers backwards at full speed for one second. The change in heading direction is achieved by a turn-in-place manoeuvre of 1.25s duration. The LGMD model reports the detection of an imminent collision when its firing rate exceeds a specific threshold, and will trigger avoidance actions until its firing rate decreases below the above mentioned threshold value.

The following analysis is based on 16 experiments in a confined environment of 3.5 by 4.5 meters that lasted approximately 3 minutes each, where both course stabilization and collision avoidance systems were active. Additionally, we performed 5 control experiments where the LGMD neuron model was active but the course stabilization system was disabled.

**Figure 6:**
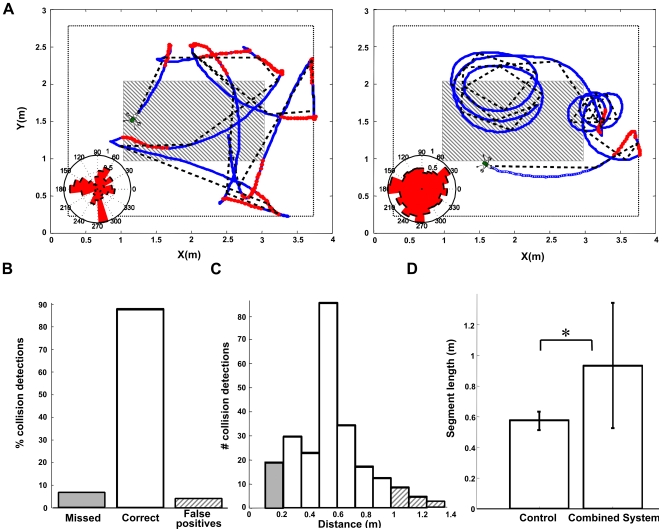
Trajectories and behavioural analysis of the “Strider” robot controlled by our LGMD model and a fly EMD based course stabilization system. (A) Representative traces of the behaviour of the robot. Left panel shows the behaviour of the robot controlled by the LGMD model in combination with an EMD-based course stabilization system, as in [37]. Right panel shows the behaviour in absence of course stabilization. The blue traces indicate the position of the robot and the red segments indicate the detection of imminent collisions. The black dashed lines are obtained by fitting linear segments to the robot traces, minimizing the Mean Square Error (MSE). Inserted into both panels are polar plots of the heading direction. (B) Collision detections between 20cm and 100cm away from the wall were classified as correct, those detected closer than 20cm from the wall (solid gray area in A) as missed, and collisions detected at a distance over 100cm as false positives (dashed area in A). (C) Detected collisions vs. distance. Bar colors correspond to the classification of the collision detection defined in panel B. (D) Segment length: Histogram of the length of the linear segments identified with the fitting procedure in (A). This measures the straightness of the traces of the robot in the control situation and when controlled by the combined course stabilization and collision avoidance system. The error bars indicate data variance. The data in (B) and (C) corresponds to 16 experiments with the combined course stabilization and collision avoidance system and 5 control experiments (LGMD alone) in (D).

The experimental results confirm the necessity of a course stabilization system: when the robot is solely controlled by the LGMD model it displayed an erratic behaviour dominated by multiple loops in either one direction or the other (Figure 6A, right panel). When the LGMD model is combined with the EMD-based course stabilization system, the robot exhibited longer periods of translation exploring a larger area, and had a less variable heading direction (Figure 6A, polar plots). The nearly uniform distribution of the variation of the heading direction during the control experiments (Figure 6A, right panel polar histogram) is the result of the continuous changes that result from the complex dynamics of the Strider robot. When both the LGMD model and the course stabilization system were combined, this distribution was significantly different and reduced to a few preferred heading directions (Figure 6A, left panel polar histogram) (p<0.01, Kolmogorov-Smirnov). To further demonstrate the effect of the course stabilization system in the control of the behaviour of the robot, a linear segmental fitting of the behavioural traces, consisting of finding a sequence of linear segments that keep the Mean Square Error (MSE) of the fit below a threshold value, was performed (Figure 6A). This measure allows quantifying the straightness of the trajectory. That is, the longer the segments are on average, the straighter the overall trajectories are (Figure 6D). In order to assess the dependency of the fit upon the threshold value, different threshold values were tested. All tested values yielded comparable results. Although it is not the objective of this study to evaluate our course stabilization model, these data serve to illustrate the complex dynamics of the Strider robot. A statistical analysis of the segment length distribution (two-sample Kolmogorov-Smirnov) showed that in case of the combined system, the distributions of the linear segments were significantly different (p<0.01). Longer segments and a higher variance were obtained for the combined system (Figure 6D); concluding that the stabilization system contributes significantly to the straightness of the trajectory. Therefore, the course stabilization system we included is an essential component in order to deal with the dynamics of the Strider, and allows us to perform and evaluate the collision avoidance task with a high-speed robot.

To evaluate the performance of the LGMD component of the robot system, all collision detections were classified into three groups: correctly detected, false negatives (missed), and false positives. These data have to be read in the context of this fast moving robot, that on average detects a collision 0.5m away from a wall while moving at a mean speed of 1.2m/s. Hence, if the robot does not dramatically change its speed at the moment of detection, it collides in less than half a second. Collisions detected 20–100cm away from the walls were considered as correct, while all collisions detected closer than 20cm from the wall were considered to be detected too late, and thus missed (false negative). Conversely, collisions detected farther than 100cm from the walls, were considered false positives (Figure 6A, grey dashed region). In total, 87.8% of the detections were correct, 4.9% were false positives and 7.3% were missed (Figure 6B). The distribution of the number of detections vs. the distance to the wall at the time of detection peaked at 0.5m, and decreased exponentially further away from the wall (Figure 6C). Thus, the behaviour of the robot directly results from the non-linear nature of the response of the LGMD model (Figure 2). Since the responses of the DCMD neuron feed directly into the thoracic motor ganglia of the locust that control the wing muscles, this seems to suggest that the amplitude-time course of the LGMD defines a particular collision avoidance strategy that minimizes the number of false positives as the distance to objects increases. In conclusion, these behavioural experiments suggest that the exponential transfer function of the LGMD neuron [9] is more related to its role in the regulation of behaviour rather than to the computation of object approach per se.

## Discussion

The question whether neurons can perform non-linear operations is of great relevance to answer what computations neuronal systems can be expected to perform. It has been argued, on the basis of the physiology of the LGMD neuron, that these neurons can perform a multiplication of high-level features of visual stimuli in order to detect pending collisions [9], [10], [16]–[18]. Gabbiani et al. proposed a model that provides for an excellent fit of the LGMD responses to looming stimuli, and as such constitutes a useful benchmark for any model of the LGMD. Using a biologically constrained model of the locust visual system, we have demonstrated that an alternative interpretation can not be excluded. In this alternative view, the local non-linear transfer function of the LGMD neuron can be accounted for in terms of the physiological and anatomical properties of its afferent visual processing hierarchy. We tested our model using simulated analogues of the locust experiments reported in the literature and assessed the real-world validity of our model using a high-speed robot. We showed that our model is able to account for all reported properties of the LGMD neuron without assuming any non-linearities other than thresholding that is intrinsic to standard leaky I&F and leaky LT neural models [32],[33] (see Materials and Methods). Consistent with our model, recent findings support the existence of a retinotopic mapping of the LGMD pre-synaptic network and suggest that a topographic map would be used to magnify the dendritic sampling of the acute zone [38]. Our model proposes an alternative view that suggests that a non-linear transfer function between stimulus and response can emerge out of the interaction of many distributed neuronal operations and their specific mapping through synaptic topologies. Moreover, our simulations show that the computation of angular speed and angular size pre-synaptic to the LGMD is not necessary to explain its properties. It has been reported that the LGMD shows an exponential relationship between the membrane potential and the firing frequency [9],[17]. Such properties are standard to integrate and fire neurons and can be explained in terms of their sigmoid transfer function [39]. As such, we believe that the LGMD has a similar transfer function and we have included it in our model. Additionally, our experiments reveal that this non-linear transfer function does not play a significant computational role in the detection of a collision, but rather that it shapes the LGMD response with respect to the behaviour requirements of collision avoidance, as demonstrated with our robot experiments.

In our analysis we have presented a plausible model of how motion selective responses can arise from the interaction between on-set and off-set sensitive neurons. The idea of having selective motion detection via delayed on-off interactions has been previously used to model visual motion-selective neurons in the mammalian neocortex [40]. The analysis of our “emergent non-linearity” hypothesis shows that the non-linear responses of the LGMD are caused mainly by the particular connectivity through the second chiasma and the parameters of the neurons in the network. It is the contribution of the restricted and local non-linearities in the medulla and structures pre-synaptic to the LGMD that give rise to the non-linear responses of our model. This mechanism is akin to the way a multilayer perceptron can approximate any continuous function with an arbitrary accuracy based on a distributed set of non-linearities [41]–[43].

Nonetheless, ours is not the first connectionist model proposed to explain the responses of the LGMD neuron to visual stimuli. In fact, Rind and Bramwell proposed a model that accounts for the looming sensitivity and selectivity when stimulated with approaching, translating or receding objects over a decade ago [44]. Consistent with ours, Rind and Bramwell's model is a feed-forward model with transient detectors (on and off-set sensitive neurons) and a feed-forward inhibition that brings the LGMD activity back to baseline. Moreover, Rind and Bramwell's model has been successfully applied to mobile robots [23]–[25],[45]. Although the model has been shown to provide a similar functionality to that of its biological counterpart, there are a number of aspects of LGMD computation that it does not account for since this model was proposed before many of the properties of the LGMD were unveiled. Thus, it does not address aspects such as the emergence of the angular threshold or the non-linear responses of the biological LGMD with respect to the specific properties of the visual stimulus (angular size and angular velocity).

Our model goes a step beyond Rind's model, making clear anatomical predictions on how the specific properties of the LGMD arise and showing that a non-linear interaction in the form of a multiplication between stimulus' angular size and velocity is not required to account for the known properties of the LGMD neuron. In our predictions, we test new stimulation protocols that would help us to better understand the functional aspects of the LGMD encoding of visual stimuli.

We have considered other possible, and probably simpler, explanations of the responses of the LGMD such as the idea that all the non-linear behaviours of this neuron could be driven directly by the input dynamics (see Text S1 for further details). Interestingly, as proposed by Rind and Simmons [11], the second derivative of the size of the looming stimulus displays a very similar time course to the actual LGMD responses. However, the second derivative model is unable to explain the invariance of the LGMD response since can not guarantee that the peak firing rate does always occur at the same angular size of the object (Figure S1). Although this stimulus dynamics based explanation cannot account for all the known LGMD properties, it does provide an alternative approach to explaining the LGMD response dynamics. To understand to what extent a direct linear mapping between input and output would suffice to explain the LGMD responses, a multivariate Least Squares linear regression method was used to fit our model's responses to a sequence of raw input images of an approaching object (Figure S1). This linear input-output mapping is indeed able to reproduce the responses of our LGMD model, as well as of its biological counterpart. Yet, as a linear mapping is not able to capture directional motion information, it fails to predict our model's responses when it was tested against receding stimuli. These two observations strongly suggest that the standard stimulation protocol used to study the LGMD neuron is under-constraint, and yields results that are insufficient to fully understand the input-output transformations it performs. In fact, what is needed are new stimulation protocols that independently manipulate both angular size and speed under different conditions – as in the linearly increasing object case – to demonstrate that the LGMD does compute the product of angular speed and size. Though some steps have been undertaken to investigate new stimulation protocols such as multiple simultaneously approaching objects, they do not capture all functional aspects of LGMD encoding of visual stimuli [31],[32],[41],[42].

Some of the stimulation protocols that we propose have recently been used in the context of the behavioral responses of the Locust to approaching predator like objects. In particular, the behavioral responses to looming and uniformly increasing angular size stimuli were studied when triggering an escape response [46]. In this study it was found that a hindleg flexion reaction (cocking) always occurred with a fixed delay after the stimulus reached a fixed angular size, independent of speed and type of approach of the stimulus. Moreover, the timing of this behavioral reaction changes in a linear fashion with the l/|v| ratio, as does the peak of the firing rate in the LGMD (Figure 2C, Figure 3). Nonetheless, there seems to be a discrepancy between these findings and the ones reported by [47], where this relationship was not found. If correct, the findings of Santer et al. would be consistent with the fact that the LGMD fires maximally when the stimulus reaches the angular threshold and thus with our predictions (see Results section). However, according to Gabbiani's model [18], the LGMD would not show a peak in its firing rate for uniformly expanding objects (Figure 5). Interestingly, sectioning the contralateral nerve cord (the stimulated DCMD) did not prevent cocking from occurring, but it just increased its variability [46]. Thus, there seem to be other parallel mechanisms that also contribute to this visually mediated behavioral response. These results seem to suggest that the role of the LGMD in this context is more related to timing of the escape action rather than the selection or execution of it.

Although there are valuable data on different stimulation protocols, there remains the need for a more detailed quantification if we want to pinpoint the underlying principle that gives rise to the non-linear responses of the LGMD. Specifically, to assess how the different parameters of different stimulation protocols (angular size and angular velocity) do affect the shape of the responses of the LGMD (the timing of the peak firing rate, the slope of the rising and declining phases, etc).

We have used our model to make functional, structural and testable predictions of the response of the LGMD. These predictions can help to explain the sub-linear behaviour found by Krapp and Gabbiani [48] when mapping the LGMD sensitivity to local motion stimuli, as well as aid in explaining the functional role of the post-synaptic inhibition. Recently, picrotoxin, a chloride channel blocker, was used to investigate the functional contribution of the feed-forward inhibition to the LGMD [35]. The main conclusion of that study was that the feed-forward inhibition contributes actively to the termination of the LGMD response to looming objects. This post-synaptic inhibition increases in an approximately exponential manner as the stimulus expands, and it is followed by a fast decay. These results are consistent and match the behaviour observed in our model. Yet, recent research has shown that other mechanisms can not be disregarded, such as spike frequency adaptation or synaptic plasticity, which can further contribute to the sharpening of the looming selectivity of the LGMD neuron [34],[39].

Finally, we implemented the LGMD model in the context of a behavioural robot experiment that demonstrates the reliability of the system to detect imminent collisions on a high-speed and inertial robot system. It has been shown that high frequency spikes of the LGMD are involved in triggering escape manoeuvres to lateral looming predators [50]. The responses of the LGMD have been shown to be correlated with cocking behavior [46], and to be sufficient to trigger gliding behavior [50]. Contrary to gliding, cocking is not necessarily triggered by the LGMD responses in isolation [46]. In fact, gliding has been shown to be triggered when the spikes of the DCMD summate significantly in the MN84 neuron, the second tergosternal flight motor neuron [50]. In this case the timing of the gliding responses is not directly related to the angular size of the visual stimulus, as in the case of cocking, but to high frequency activity (>150Hz) produced by the LGMD neuron. The difference between relying on the angular size of the approaching object or on high frequency activity from the LGMD supports the notion that gliding is triggered as a “last resort” when the other existing mechanisms to evade a thread fail [51],[52]. In the case of our high-speed robot experiments we have used a very similar approach to what occurs in gliding. That is, the robot only triggers an avoidance reaction when the responses of the LGMD summate over a threshold in a motor neuron responsible for the avoidance reactions (see Robot Experiments section).

Furthermore, our experiments show that the exponential transfer function of the LGMD could play an important role in minimizing the probability of false positive detection at long distances from obstacles without compromising the performance of the system. We thus propose that the exponential Vm to firing rate mapping of the LGMD may more be related to its role in the regulation of behaviour than to its putative computational role in input processing.

## Materials and Methods

In the evaluation of our model we employed a twofold strategy: On the one hand, we characterized our model using protocols identical to those reported in the literature, i.e. approaching stimuli with different speeds, shapes and textures, which were displayed on a LCD screen and captured with a CCD camera. Additionally, new stimulation protocols were used to make predictions of the responses of the biological system. On the other hand, we studied the behavioural implications of our model by applying it to a high-speed robot.

### Stimulation protocols

The rate of expansion of an approaching object of half length *l* with velocity *v* was reproduced by a simulated looming stimulus. Any object approaching at a constant speed shows a typical slow angular speed that rapidly increases as it gets closer to the camera. The angular size of this approaching object can be described as a function of *l* and *v*, where *l* is the half-size of the object length and *v* its linear velocity.
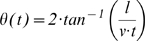
(3)Consistent with previous studies (Gabbiani et al., 2002; Gabbiani et al., 1999; Gabbiani et al., 2001), looming stimuli with *l/|v|* ratios that range from 5 to 50ms, with a 5ms step size, were used, with 10 to 21 repetitions for each stimulation condition. Using these stimuli we have assessed the relationship between the responses of the model LGMD and stimulus properties, including the relationship between the TTC and the *l/|v|* ratio and the invariance of the angular threshold of the LGMD response over the whole range of *l/|v|* ratios used. In these experiments the stimuli are presented as a solid shape (square) and the centre of the screen is aligned with the centre of the camera in both azimuth and elevation. Subsequently, we performed a set of measurements in order to establish the dependence of the LGMD response on the shape and texture of the stimulus using stimuli reported in the literature: a solid square, a solid circle, a square with a checkerboard pattern and a square with a pattern consisting of concentric squares [17]. Finally, we investigated the invariance of the responses of the LGMD model to the approach angle considering presentation angles of stimuli corresponding to 0%, 33%, 55%, and 75% of the visual field of the camera, where 0% represents the alignment of the camera with the screen, and at 100% the looming stimulus lies outside of the visual field of the CCD camera.

A high-end CCD camera (EVI-D31, Sony Corp., Japan) placed 10cm in front of the screen was used as input to our model. The camera was positioned such that its image covered the complete display, resulting in a visual field size of 74.65°H×56.25°V. To present the looming stimuli, a LCD screen with a resolution of 800×600 pixels was used. The spatial resolution of the screen (0.019cm per pixel) corresponds to an angular resolution of 0.0933° per pixel. The highest luminance (L_High_) value reported by our video acquisition system (mean of value for the RGB color channels) was defined as 255 and the lowest as 0 (L_Low_) on a 0 to 255 scale. The stimuli were generated with an ideal luminance contrast 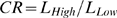 of infinity, where CR = 1 indicates no contrast. The acquisition rate of the camera was 25Hz (PAL) and the refresh-rate of the LCD monitor was set to 60Hz. For the purpose of the simulations presented here, the system was not required to run in real-time. Thus, we simulated a processing power of 100 images per second for our model. For the acquisition, we approximated a uniformly distributed compound eye of 32×24 ommatidia/photoreceptors. This is obtained by sub-sampling the image that is acquired from the camera, making the step size increase of the looming stimulus negligible. The resulting angular resolution corresponds to 2.33° per pixel, a good match to the real photoreceptor acceptance angle of the locust which is close to 1.5° in light conditions and 2.5° when dark adapted [36].

### Robot, arena and tracking system

**Figure 7:**
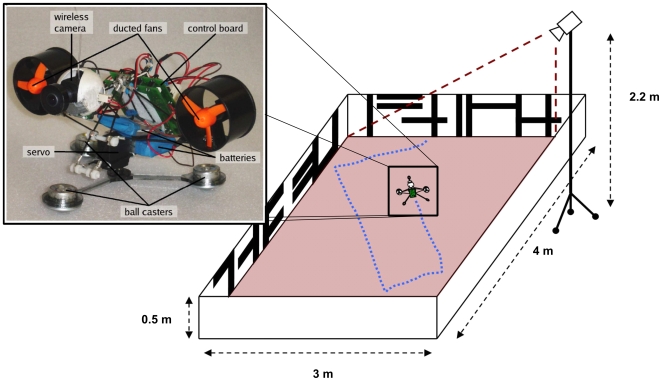
The insect robot and its test arena. Left panel: The “Strider” robot with its components. Right panel: Schema of the arena used to test the LGMD model including the “AnTS” tracking system setup. See text for further explanation.

We evaluated the behavioural implications of our model using a ball caster based robot platform called “Strider”, specifically designed to have low frictional forces with the surface and that uses a propulsion system that allows it to deliver high-speeds, with the advantage of a low deployment and maintenance effort (Figure 7, left panel). The Strider is about 16cm long and it is equipped with three passive wheels (ball casters) (Euro Unit 15mm, AlwayseEngineering Ltd, United Kingdom), and propelled by two ducted fans (GW/EDF-50, Grand Wing Servo-tech Co., Ltd., Taiwan). The base platform on which the wheels are mounted connects to the upper part via a servo (Microservo FS 500 MG, Robbe Modellsport GmbH & Co, Germany), allowing the robot to turn in place, a task difficult to achieve with ducted fans alone. The lift-strength of one ducted fans is 30g, allowing the robot to move at a maximum speed of about 3m/s which corresponds to 19 body lengths per second. Similarly, the locust displays a free flight speed of about 4m/s [53].

Two separate lithium-polymer batteries (t-technik, Germany) are used as independent power-supplies for controller-board and sensors, and motors respectively. The total weight of the robot is 280g. A Bluetooth® link is used to send control signals to the motors of the robot and to read sensor states from the robot. The robot carries a wireless camera (1.2GHz Mini Wireless Camera Kit, ZTV Technology Co., Ltd, China) with a 190° wide-angle lens.

The robot experiments were performed in a 3×4m arena (Figure 7, right panel). The walls of the arena (0.5m high) were covered with random textures consisting of vertical and horizontal stripes to provide the robot with visual cues. The behavioural data was acquired in real-time with a custom-built general purpose video tracking system called “AnTS” developed by the authors. The AnTS tracking system receives its input from a B/W CCIR camera (CSB-465C, Pacific Corporation, Japan) with a wide-angle lens fixed on a 2.2m high tripod. To obtain an undistorted planar view of the arena, correction algorithms for perspective and wide-angle lens distortions were built into the AnTS tracking software. As a compromise between sampling frequency and spatial accuracy, a QVGA image resolution (320×240 pixels) was used; this resulted in a spatial resolution of 1.56cm for the 3×4m arena and an update frequency of 35Hz. The behavioural data recorded with AnTS was acquired synchronously with the states of the model of the locust visual system (see below).

### Dynamics of the neuron models

Two standard neuron types are used in these simulation experiments: Leaky Integrate & Fire (I&F) and leaky Linear Threshold (LT) neurons [32],[33]. Both neuron models are equivalent to a circuit built from a capacitor *C* and a resistor *R* connected in parallel to ground on one end and driven by current on the other end [39]:
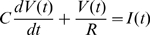
(4)For a constant input current the voltage is defined by:
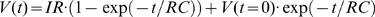
(5)The voltage at the membrane of both neural models will increase asymptotically to 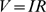. While the voltage is below the firing threshold (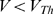) the neuron remains silent, and once 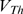 is reached the neuron's output is equal to the membrane potential in the case of LT, or it produces an action potential (spike) and resets the membrane voltage 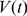 to zero in the case of the I&F. The charging time constant of the membrane potential is defined as 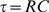.

### Model

Our model captures the basic processes found in the locust visual system and can be divided into three sequential processing steps (Figure 1). First, the centre-excitation/surround-inhibition connectivity among the signals received from the photoreceptors in the lamina layer that provides an edge enhancement [31]. Second, the interaction of neurons in the medulla layer yields onset and offset sensitive responses [13]–[15]. Third, the lobula layer provides a specific connectivity that contributes to the transformation of the onset/offset signals into the response of the LGMD. Our model is structured exclusively with leaky Integrate and Fire (I&F) and leaky Linear Threshold (LT) neurons (see Experimental Procedures for the dynamic equations) and implements the three layers described above.

**Figure 8:**
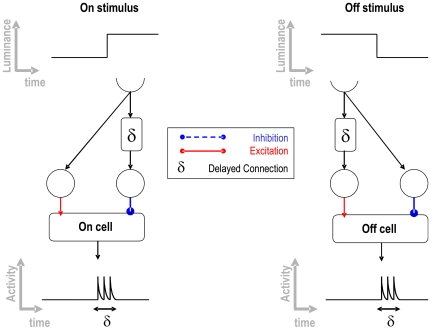
Neural connectivity that accounts for the on-off sensitive cell responses. This network makes use of the interaction of delayed and non-delayed excitatory and inhibitory pathways.

An edge enhancement on the input image is achieved via a centre-excitation/surround-inhibition connectivity from the photoreceptors to the lamina layer, modelled as LT neurons. Our model implements onset and offset responses of the medulla by combining the activity of one excitatory and one inhibitory neuron with the same visual sensitivities from the lamina onto a common third neuron, where the inhibition is time delayed relative to the excitation in case of onset detection, and time advanced relative to the excitation in case of offset detection (Figure 8).

When we assume that a transition of activity in the receptive fields of the on and off neurons is a moving edge, there exists a unique arrangement of on and off cells with a combined response that is maximal whenever the moving edge is being displaced in a specific direction, i.e. neighbouring cells placed along the movement axis, where a first offset sensitive cell and a second onset sensitive cell synapse onto a common neuron. The post-synaptic neuron is maximally excited only when both pre-synaptic cells are active at the same time, i.e. when an offset and an onset stimulus coincide. Hence, the only situation that can provoke this kind of response is a moving edge passing out of the off cell's receptive field, generating an offset event, to the receptive field of the on cell, generating an onset response. Therefore, as outlined above, a pair-wise combination of on and off transient detectors can encode for directionally selective motion pre-synaptic to the LGMD. This neuronal processing structure is consistent with our knowledge of the pre-synaptic structure of the LGMD since the 1970s [13]–[15].

**Figure 9:**
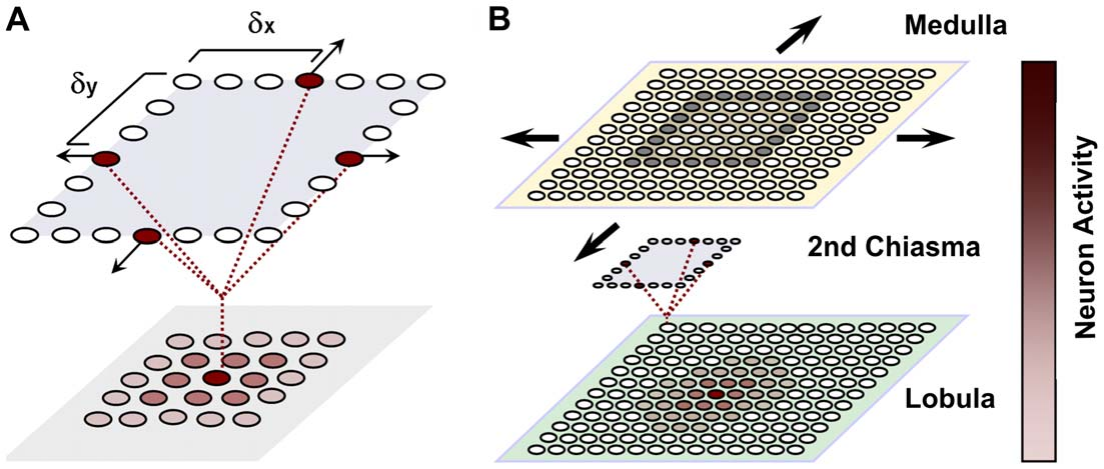
Schema of the connectivity between the medulla and the LGMD pre-synaptic fan. (A) Connectivity pattern for each of the neurons connecting medulla and lobula that mediate the excitatory pathway to the LGMD. In the case of our model, the angular threshold is defined by the size of the surrounding excitation (red neurons), where the edges of a looming object would sit to provide the maximum excitation (δx, δy). (B) Example of how a looming square stimulus would excite the on-off neurons in the medulla and the pre-synaptic excitatory fan of the LGMD. The activity is color-coded. The cells placed in the centre of the object maximally excited during the approach movement, and poorly otherwise.

One of the most important and most studied properties of the LGMD is related to the angular threshold (θ_threshold_), which is defined as the angular size of a looming object for which the LGMD produces the maximal firing rate. It has been shown that there is a constant relationship between the peak firing rate of the response of this neuron and the angular size of the looming stimulus, independent of the approach speed and angle, object shape, texture and contrast [9],[16],[17]. To account for the angular threshold properties (θ_threshold_) we propose a specific connectivity between the on-off cell ensembles onto the LGMD, referred to as the LGMD pre-synaptic fan (Figure 9). It is central to our hypothesis that the projections from the medulla to the lobula are such that the excitation on the target cells is maximal when the collection of detected oriented contrast boundaries reach a specific size. The LT neurons connecting the medulla with the LGMD through the second chiasma collect the activity of a set of surrounding on-off neurons in the medulla with a particular directional selectivity at distances δx and δy (Figure 9). These LT neurons have lateral interactions with the neighbouring cells via a lateral excitation that spreads and smoothes their activity over the pre-synaptic excitatory fan of the LGMD (Figure 9A). The δx and δy define the width and height of the connectivity where the expanding boundaries of a looming stimulus lie to maximally excite that post-synaptic neuron. This connectivity pattern is applied to each of the neurons that mediate the excitatory pathway to the LGMD across the second chiasma and receive input from the onset/offset sensitive cells. These neurons will concentrate a spot of high activity for looming stimuli approaching the angular threshold size whereas a sparse distribution of activity will occur for other stimuli (receding, translating, etc) (Figure 9B).

It is now possible to define the exact values of δx and δy that make the LGMD maximally excited for a given object angular size and excited below maximum otherwise. The accuracy with which we can define the angular threshold is given by the resolution of our model, being ±2 • acceptance angle of one pixel (approximately ±5°).

In our implementation of the model, only four type of ensembles of on and off neurons with different directional sensitivities were used (Figure 9A). By means of a thresholding mechanism, the LT neurons that cross the second chiasma respond only when a number of the surrounding (δx and δy) pre-synaptic motion sensitive ensembles detect expanding moving edges. Hence, by looking at the neural activity of this layer of neurons it is possible to extract the position of the looming stimulus in the visual field (Figure 9B). Subsequently, the spatial integration by the LGMD pre-synaptic fan of those responses discards the position information, and in this way introduces the important property of response invariance to object position and approach angle. The structure of the feed-forward network up to this point supports the consistency and invariance of the angular threshold (θ_threshold_), i.e. the independency of the approach angle, position inside the visual field, object shape and looming speed. In the last processing stage, the LGMD receives a post-synaptic inhibition from the activity of the on-off neurons in the medulla (Figure 8). The role of this inhibition is to bring the LGMD neuron's activity back to baseline after the looming object reaches the angular threshold size.

For the data analysis, a Gaussian smoothing filter with a window size of 20ms was applied to our raw data, consistent with previous LGMD studies [9]. The membrane potential of the LGMD was computed in the simulation while the used Vm/F transfer function of the LGMD neuron is consistent with the one reported in the literature [16],[17]. A one-way ANOVA analysis was used to evaluate significant differences between the data sets obtained during the experiments.

### Simulation

The simulations were performed on a 2GHz Pentium4 personal computer (Intel, Santa Clara, USA) under the Linux operating system. The neural simulation software **iqr**, an open source simulation software (iqr.souceforge.net), was chosen for the implementation and evaluation of the neural model, including the robot experiments [54]. All creation of visual stimuli was performed using openCV (the Open Source Computer Vision library, Intel, Palo Alto, USA) while the analysis was performed using Matlab (Mathworks, Natick, Massachusetts, USA).

## Supporting Information

Text S1 Model analysis and further model comparisons
(0.11 MB DOC)

Figure S1 Comparison of the observed parameter space of the LGMD responses with two alternative models. Second derivative of the angular size of a looming stimulus (gray solid line). A multivariate linear regression was used to fit our model's responses to a raw sub-sampled sequence of images input to the system (16×14 pixels) (dashed red line). All the model responses were normalized for the maximum firing rate for comparison purposes. See text for further information.
(1.43 MB TIF)
