# Supplementary material for: Non-Linear Neuronal Responses as an Emergent Property of Afferent Networks: A Case Study of the Locust Lobula Giant Movement Detector
Source: PLoS Comput Biol. 2010 Mar 12;6(3):e1000701. doi: 10.1371/journal.pcbi.1000701 (PMC2837398; doi:10.1371/journal.pcbi.1000701)
Supplement: Text S1 — Model analysis and further model comparisons (0.11 MB DOC) [file pcbi.1000701.s001.doc]

# Model analysis

In order to identify the processes and the contribution to the LGMD’s responses of the individual layers of our model, an analysis of the system was conducted. The neural mean population responses of all the layers were analyzed with standard and novel stimulation protocols. To understand the processing of our system, the model is decomposed into its 5 different layers (Figure 1). In the following, we make a functional analysis of the different layers of the model only taking the essential cell parameters that give rise the given functionality.

At the photoreceptor level (Figure 1A), the mean activity corresponds directly to the activity generated by the visual input. For a black looming stimulus on a white background it corresponds to:

$\begin{matrix} Mean_{Photoreceptor}(t)=\frac{1}{N_{x}N_{y}}\sum_{x}^{N_{x}} \sum_{y}^{N_{y}} a_{Photoreceptor}(x,y,t)= \\ \frac{1}{N_{x}N_{y}}\left(N_{x}N_{y}\cdot White_{baseline}-const\cdot\theta^{2}(t)\right)=White_{baseline}-k\cdot\theta^{2}(t) \end{matrix}$ (6)

where$a_{photoreceptor}(x,y,t)$ represents the activity of the photoreceptor at position (x, y) at time *t.* $N_{x}$and $N_{y}$define the number of neurons of this layer in *x* and *y* coordinates, *k* is a constant and $White_{baseline}$is the photoreceptor’s response to stimulation with white light. The number of photoreceptors that are covered by the object is determined by the area of the object on the retina, i.e. $\propto\text{ }\theta^{2}(t)$. This is reflected in the responses for the three stimulation protocols used, simulated approaching object, uniformly increasing object size and simulated receding object (Figure 1A, Figure 5A). A simulated approaching object shows a greater decrease at the last part of the stimulation, whereas the uniformly increasing object shows it from the beginning (Figure 1A, Figure 5A).

At the level of the lamina, an edge enhancement is performed using centre-excitation/surround-inhibition connectivity (Figure 1B, Figure 5B), equivalent to a convolution operation of the visual input with a difference of Gaussian kernel [1]:

$\begin{matrix} a_{\text{lamina}}(i,j,t)=a_{Photoreceptors}*DiffGauss_{Kernel}= \\ \sum_{x}^{N_{x}} \sum_{y}^{N_{y}} a_{Photoreceptor}(x,y,t)\cdot DiffGauss_{Kernel}(i-x,j-y) \end{matrix}$ (7)

where $a_{\text{lamina}}(x,y,t)$ is the activity of the laminar neuron at the position (x, y) at time *t* resulting from the convolution operation, and $DiffGauss_{Kernel}$ is a difference of Gaussians kernel.

$\begin{matrix} DiffGauss_{Kernel}(x,y)=gain_{1}\cdot f(x,y,\sigma_{1})-gain_{2}\cdot f(x,y,\sigma_{2}) \\ f(x,y,\sigma)=\frac{1}{\sqrt{\text{2π}}\sigma}\text{exp}\left(-\frac{x^{2}+y^{2}}{\text{2σ}^{2}}\right) \end{matrix}$ (8)

In this case, the 2-dimensional Gaussians are centered at (0,0), with standard deviation values $\sigma_{1}>\sigma_{2}$. Effectively, the result of this linear operation corresponds to an edge enhancement. Therefore, the mean activity of this layer $(Mean_{Lamina})$ can be approximated as being directly proportional to the edges of the looming stimulus, i.e. $\propto\text{ }\theta(t)$ (Figure 1B, Figure 5B).

$Mean_{\text{Lamina}}(t)=\frac{1}{N_{x}N_{y}}\sum_{x}^{N_{x}} \sum_{y}^{N_{y}} \left[a_{Photoreceptors}*DiffGauss_{Kernel}\right]\approx k\cdot\theta(t)\begin{matrix} \\ \end{matrix}$ (9)

The on-off neurons compute the difference between delayed inputs, i.e. a temporal difference with a time step equal to the connection delay ($\Delta t=\delta$) (Figure 8). Indeed, this means that the aggregation of on-off neurons will only fire when the motion is produced in the preferred direction, as described in the Experimental Procedures. The effect is that only moving edges in a radial outward fashion will be detected in the Medulla, whereas still edges will not elicit responses. The result of this process (in the case of a looming object) is a response directly proportional either to $\theta(t)$ when looming and 0 when no motion is present. In the case of I&F neurons stimulated by motion respond with an action potential of fixed amplitude. The mean activity would then be directly proportional to the number of neurons stimulated, i.e. $\propto\theta(t)$ while looming:

$$Mean_{on-off}(t)=\frac{1}{N_{x}N_{y}}\sum_{x}^{N_{x}} \sum_{y}^{N_{y}} \left[a_{Photoreceptors}*DiffGauss_{Kernel}\right]=\frac{1}{N_{x}N_{y}}\sum_{x}^{N_{x}} \sum_{y}^{N_{y}} a_{on-off}(x,y,t)\approx\text{ }$$

$\approx\left(\begin{matrix} (A) & k\cdot\theta(t) & \text{if looming} \\ & & \\ (B) & 0 & \text{if no motion} \end{matrix}\right.$ (10)

The effect of the computation of the on-off neurons in the responses of this layer results in a null response when there is no motion in the stimulus, i.e. the stimulus is bigger that the field of view (Figure 1C, Figure 5C), being consistent with eq. 10B. However, when an approaching object is shown, the response follows linearly the input, according to eq. 10A (Figure 1C, Figure 5C, left panel). This is the first important non-linearity that emerges from the specific topology and neural parameters of the model, and that doesn’t allow for a concise mathematical formulation of the LGMD’s responses.

The connectivity with the on-off neurons (Figure 9A) together with the thresholding mechanism of their postsynaptic LT neurons play a crucial role in our model mediating the excitatory input to the LGMD. These two parameters, in combination with the lateral connectivity between the neurons pre-synaptic to the LGMD, cause the mean activity of the excitatory pathway to the LGMD to linearly follow the input activity for object sizes up to the angular threshold (θ_threshold_) (Figure 9A). This phenomenon occurs solely for looming stimuli due to the specific connectivity with the on-off aggregations that respond to specifically oriented motion, i.e. in a radial outward fashion. After the θ_threshold_ is reached, the size of the object is bigger than the surrounding connectivity pattern (Figure 9A) and thus the excitatory input reaches a plateau, and decreases thereafter as the looming stimulus covers the whole visual field. This is the most important non-linearity of the entire system, and it emerges from a distributed thresholding mechanism, which is crucial to explain most of the properties reported in this paper (see Methods section for further details on the connectivity pattern). Therefore, in the case of looming stimuli we find:

$LGMD_{Exc}(t)=\sum_{x}^{N_{x}} \sum_{y}^{N_{y}} Thr\left(a_{on-off}(x,y,t)*Connec_{Kernel}(x,y)\right)\approx$

$\approx\left\{\begin{matrix} \begin{matrix} \begin{matrix} \begin{matrix} k\cdot\theta(t)\text{ }\theta(t)<\theta_{threshold} \\ \end{matrix} \\ k\cdot\left(2\cdot\theta_{threshold}-\theta(t)\right)\text{ }\theta_{threshold}<\theta(t)<\frac{3\cdot\theta_{threshold}}{2} \end{matrix} \\ \end{matrix} \\ k\cdot\frac{\theta_{threshold}}{2}\text{ }\theta(t)>\frac{3\cdot\theta_{threshold}}{2} \end{matrix}\right.\text{ if looming}$ (11)

where the excitatory input to the LGMD neuron $LGMD_{Exc}$ is the result of applying thresholding operation *Thr* to the convolution of the activity of the on-off neurons with a connectivity kernel $Connec_{Kernel}$(eq. 11) (Figure 9A).

The post-synaptic inhibition to the LGMD collects the overall activity of the on-off neurons located in the medulla (eq. 11). Therefore, the difference between excitation and inhibition will basically approximate the membrane potential of the LGMD neuron$(LGMD_{Vm})$, which will be subsequently mapped into firing rate:

$LGMD_{Inh}(t)=Mean_{on-off}(t)=k\cdot\theta(t)\text{ if looming}$ (12)

$$LGMD_{Vm}(t)=gain_{Exc}\cdot LGMD_{Exc}(t)-gain_{Inh}\cdot LGMD_{Inh}(t)=$$

$\approx\left\{\begin{matrix} \begin{matrix} \begin{matrix} \begin{matrix} k\cdot\left(gain_{Exc}-gain_{Inh}\right)\cdot\theta(t)\text{ }\theta(t)<\theta_{threshold} \\ \end{matrix} \\ k\cdot\left(gain_{Exc}\cdot\left(2\cdot\theta_{threshold}-\theta(t)\right)-gain_{Inh}\cdot\theta(t)\right)\text{ }\theta_{threshold}<\theta(t)<\frac{3\cdot\theta_{threshold}}{2} \end{matrix} \\ \end{matrix} \\ k\cdot\left(\frac{\theta_{threshold}}{2}\text{ }-gain_{Inh}\cdot\theta(t)\right)\text{ }\theta(t)>\frac{3\cdot\theta_{threshold}}{2} \end{matrix}\right.\text{ if looming}$ (13)

$LGMD_{Firing}=\exp\left(LGMD_{Vm}\right)=\exp\left(LGMD_{Exc}-LGMD_{Inh}\right)$ (14)

where $gain_{Exc}$and $gain_{Inh}$define the contribution of each of them to the LGMD responses.

It has to be remarked that in the here above formulation only the key structural elements have been captured (connectivity and neural non-linear - thresholding - operations), and in order to make a direct comparison of these theoretical responses of our model with the ones observed in its biological counterpart, the values for the neurons’ parameters would have to be considered (membrane capacitance, refractory period, synaptic weights, etc). Nevertheless, an analytical formulation of such a complexity becomes impracticable, and it is indeed the reason of our numerical simulation study.

# Extended comparison of the LGMD responses

The parameter space of the fits used in [2] was considered for a comparison with two alternative models. Gabbiani et al. used two additional parameters to fit eq. 1 to the obtained data, $N_{1}$and$N_{2}$.These are real power exponents fitted to the rising and decaying phase of eq. 1, and typical values used for$N_{1}$range from 1.0 to 3.0, and from 0.1 to 2.0 in case of$N_{2}$:

$fit=\left\{\begin{matrix} (eq.1)^{N_{1}}\text{ t}\leq\text{t}_{\text{peak}} \\ \\ (eq.1)^{N_{2}}\text{ t}>\text{t}_{\text{peak}} \end{matrix}\right.$ (15)

In addition to the analysis of our model, we also considered two alternative ways of explaining the LGMD responses. Firstly, we investigated the idea that all the non-linear behaviors of this neuron are directly driven by the input dynamics. In this case we found that the acceleration of the looming stimulus (second derivative of eq. 3), as proposed by Rind and Simmons [3], displays a very similar time course to the actual LGMD responses (Figure S1A). Nevertheless, as opposed to the LGMD neuron and the model proposed here, a further investigation of the second derivative hypothesis shows that the peak firing rate varies in a non-linear fashion relative to the TTC:

$\theta''(t)=\frac{4l\cdot\csc\left(\frac{l}{t\cdot v}\right)^{3}\left(l\cdot\cos\left(\frac{l}{t\cdot v}\right)-t\cdot v\cdot\sin\left(\frac{l}{t\cdot v}\right)\right)}{t^{4}\cdot v^{2}}$ (16)

In other words, the peak firing rate of this model, *max {$\theta''$}*, does not occur always at the same object’s angular size. Thus, this model is unable to explain at least the $\theta_{threshold}$ invariance property. Despite the fact that it cannot account for all the known LGMD properties, it provides a new approach for explaining the LGMD response dynamics in an entirely linear way.

Subsequently, we wanted to assess whether a linear mapping input-output would suffice to explain the LGMD responses. That would mean that in theory, there would exist a direct mapping from the ommatidia to the LGMD that would account for the data. To test this hypotheis, a multivariate Least Squares linear regression method was used to fit our model's responses to a sequence of raw subsampled input images (16 x 14 pixels) during stimulation of our system with looming objects. This method defines a linear input-output mapping in a single layer that minimizes the error between the data and the regression (Figure S1B). This investigation revealed that simply by taking into account the characteristics of the visual input, a simple weighing of the response of the photoreceptors is able to reproduce the responses of our LGMD model for a looming object, as well as of its biological counterpart. On the other hand, the linear mapping was not able to predict our model’s responses when the linear regression was tested against receding stimuli since directional motion information is not taken into account by such a linear model.

# References

1. Gonzalez, R. and R. Woods, Digital Image Processing. 1992: Addison-Wesley Publishing Company. 442.

2. Gabbiani, F., et al., Multiplicative computation in a visual neuron sensitive to looming. Nature, 2002. **420**(6913): p. 320-4.

3. Rind, F.C. and P.J. Simmons, Orthopteran DCMD neuron: a reevaluation of responses to moving objects. I. Selective responses to approaching objects. J Neurophysiol, 1992. **68**(5): p. 1654-66.
