# Supplementary figures and images for: Non-Linear Neuronal Responses as an Emergent Property of Afferent Networks: A Case Study of the Locust Lobula Giant Movement Detector
Source: PLoS Comput Biol. 2010 Mar 12;6(3):e1000701. doi: 10.1371/journal.pcbi.1000701 (PMC2837398; doi:10.1371/journal.pcbi.1000701)

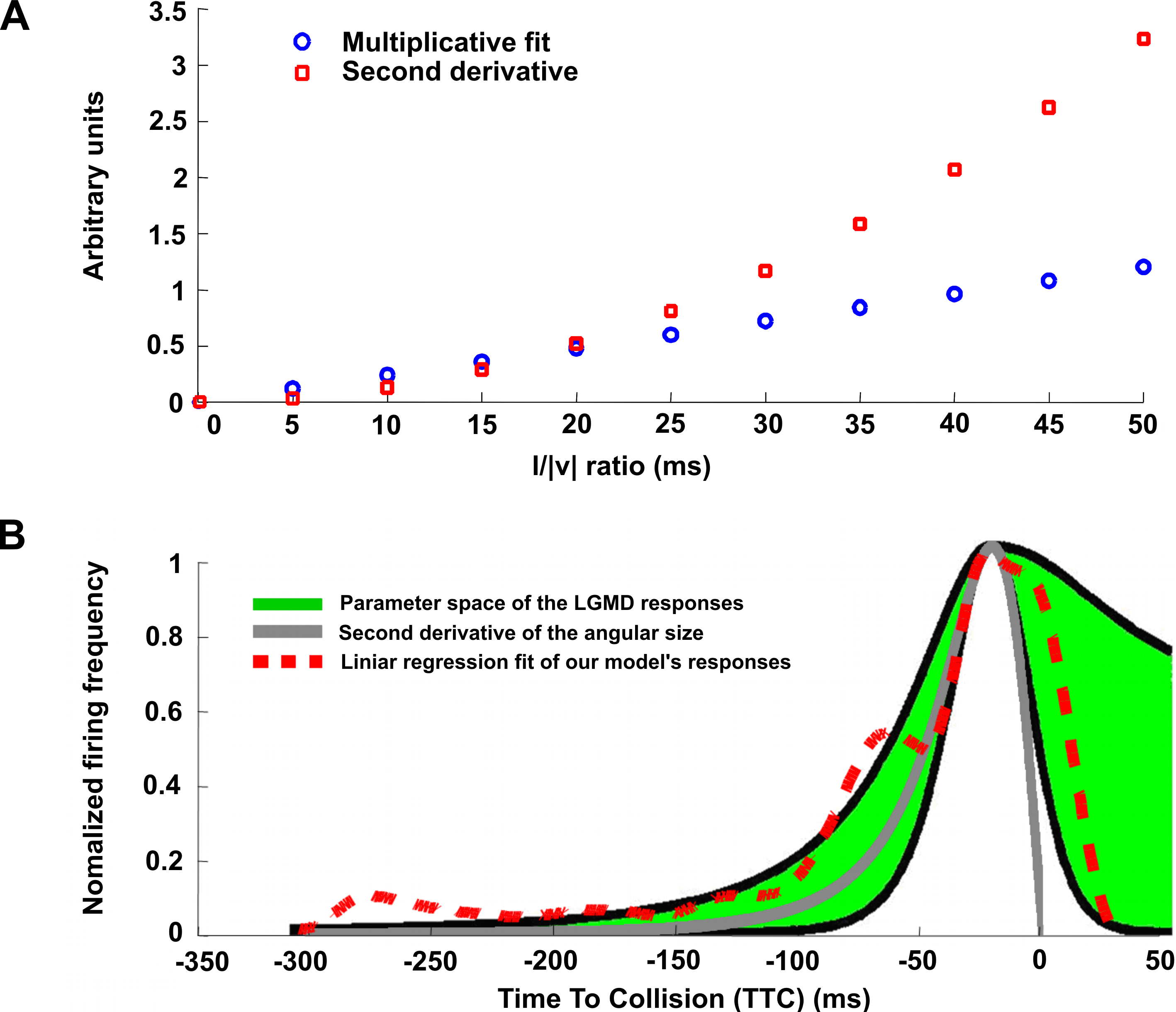

Supplement: Figure S1 — Comparison of the observed parameter space of the LGMD responses with two alternative models. Second derivative of the angular size of a looming stimulus (gray solid line). A multivariate linear regression was used to fit our model's responses to a raw sub-sampled sequence of images input to the system (16×14 pixels) (dashed red line). All the model responses were normalized for the maximum firing rate for comparison purposes. See text for further information. (1.43 MB TIF) [file pcbi.1000701.s002.tif]
